# Gui-zhi-fu-ling-wan alleviates bleomycin-induced pulmonary fibrosis through inhibiting epithelial-mesenchymal transition and ferroptosis

**DOI:** 10.3389/fphar.2025.1552251

**Published:** 2025-04-16

**Authors:** Zi-Yong Chen, Meng-Meng Ma, Rui Wang, Qing-Qing Zhang, Mei-Ling Xie, Ying-Li Wang, Yong-Xia Guo, Kui Liu, Li-Fang Cao, Feng-Lian He, Lin Fu, Ya-Lin Jiang

**Affiliations:** ^1^ The Affiliated Bozhou Hospital of Anhui Medical University, Bozhou, Anhui, China; ^2^ Department of Respiratory and Critical Care Medicine, The Affiliated Bozhou Hospital of Anhui Medical University, Bozhou, Anhui, China; ^3^ Department of Respiratory and Critical Care Medicine, The Second Affiliated Hospital of Anhui Medical University, Hefei, Anhui, China; ^4^ Institute of Respiratory Diseases, The Second Affiliated Hospital of Anhui Medical University, Hefei, Anhui, China

**Keywords:** gui-zhi-fu-ling-wan, pulmonary fibrosis, bleomycin, epithelial-mesenchymal transition, ferroptosis, mitochondrial reactive oxygen species

## Abstract

**Background:**

Idiopathic pulmonary fibrosis (IPF) has a higher morbidity and poor prognosis. Gui-Zhi-Fu-Ling-Wan (GFW) is a traditional Chinese herbal formula which exerts anti-inflammatory and anti-oxidative effects. The goal was to determine the protective effect of GFW on bleomycin (BLM)-induced pulmonary fibrosis.

**Methods:**

One hundred and twenty-four mice were randomly divided into eight groups, and orally supplemented with GFW (1 g/kg) in 1 week ago and continuing to 1 week later of single BLM intratracheal injection (5.0 mg/kg). Lung tissues were collected in 7 days and 21 days after BLM injection. BEAS-2B cells were pretreated with GFW (100 μg/mL) for three consecutive days before BLM (10 μg/mL) exposure. Cells were harvested in 12 or 24 h after BLM co-culture.

**Results:**

GFW supplementation alleviated BLM-induced alveolar structure destruction and inflammatory cell infiltration in mice lungs. BLM-incurred collagen deposition was attenuated by GFW. In addition, GFW pretreatment repressed BLM-evoked downregulation of E-cadherin, and elevation of N-cadherin and Vimentin in mouse lungs. Besides, BLM-excited GPX4 reduction, ferritin increases, lipid peroxidation, and free iron overload were significantly relieved by GFW pretreatment in mouse lungs and BEAS-2B cells. Notably, BLM-provoked mitochondrial reactive oxygen species (mtROS) excessive production, elevation of mitochondrial stress markers, such as HSP70 and CLPP, and mitochondrial injury, were all abolished in mouse lungs and BEAS-2B cells by GFW pretreatment.

**Conclusion:**

GFW supplementation attenuated BLM-evoked lung injury and pulmonary fibrosis partially through repressing EMT and mtROS-mediated ferroptosis in pulmonary epithelial cells.

## 1 Introduction

Idiopathic pulmonary fibrosis (IPF) is a chronic and progressive lung disease of unknown origin with a bad prognosis (Richeldi et al., 2017). It has a natural incidence related to age and a high mortality rate (Nalysnyk et al., 2012). The median survival period after diagnosis is only two to 3 years (Wolters et al., 2014). The incidence of pulmonary fibrosis is estimated to be around 3–9 cases per 100,000 people in Europe and North America, which is relatively lower compared to Asia (Borchers et al., 2011). In the present, there are very few treatment measures. The effectively anti-fibrosis drugs include bifenidone and nidanib, which can slow down the decline in pulmonary capacity, but fail to improve prognosis and reverse the progression of IPF (Ding et al., 2023) and also have a host of vice effects (Zhai et al., 2023). Lung transplantation is currently the only curative treatment for IPF. However, the limited availability of suitable donor organs and the relatively short post-transplant survival time pose the challenges to the widespread application of lung transplantation (George et al., 2019). Therefore, exploring new treatment methods and drugs are needed urgently for IPF.

Gui-Zhi-Fu-Ling-Wan (GFW) is a traditional Chinese herbal formula that has been utilized in a variety of gynecological illnesses (Zheng et al., 2020), consisting of dysmenorrhea and menstrual irregularities. Its primary constituents comprise of Gui Zhi (Cinnamomum cassia), Fu Ling (Poria cocos), Tao Ren (Prunus persica), Mu Dan Pi (Paeonia suffruticosa), and Shao Yao (Paeonia lactiflora), known for their multifaceted therapeutic effects such as improving blood flow, resolving inflammation, and alleviating pain (Liu et al., 2024). The previous study have provide compelling evidence suggesting that GFW has the potential to alleviate liver fibrosis (Liu Z. et al., 2022). Additionally, it has been shown that GFW can effectively inhibit fibrobactivation and exert an anti-oxidative stress effect (Yao et al., 2024). Therefore, we speculated that whether GFW has certain curative effect for IPF.

The mounting evidence has revealed that epithelial-mesenchymal transition (EMT), ferroptosis, and mitochondrial oxidative stress exert vital roles in the pathological process of pulmonary fibrosis (Li M.-D. et al., 2024; Li et al., 2024 S.-R.). Pharmacokinetic analysis indicated that GFW primarily contains a lot of key active ingredients such as paeoniflorin, gallic acid, amygdalin, cinnamic acid, salvinorin, and paeonilactone glycosides (Zhao et al., 2015). The earlier studies have hinted that paeoniflorin can inhibit transforming growth factor-beta (TGF-β)-induced EMT (Ji et al., 2016) and carbon tetrachloride-mediated ferroptosis (Zhao et al., 2015). Moreover, Gallic acid, known for its antioxidant properties, has been found to induce apoptosis and inhibit fibrosis in pulmonary fibroblasts (Chuang et al., 2010). Besides, it is also shown that Gallic acid is able to attenuate lung fibrosis induced by BLM through antioxidant effect (Rong et al., 2018). Furthermore, it is reported that a complex of cinnamic acid and flavonoids effectively alleviative mitochondrial stress and ferroptosis induced by erastin (Günther et al., 2023). Consequently, the objective was to assess the impact of GFW supplementation on BLM-induced pulmonary fibrosis in mice and to explore the underlying mechanisms. These results provide significant evidence that GFW has protected against BLM-induced pulmonary fibrosis. This study may offer clinical evidence and laboratory insights in the future treatments for pulmonary fibrosis.

## 2 Materials and methods

### 2.1 Animals and treatments

Adult male C57BL/6J mice (8 weeks old, 21–24 g) were bought and housed at the Specific Pathogen Free (SPF) animal laboratory. The study included two separate animal experiments.

(1) In Experiment one, to investigate the protective effects of GFW against ferroptosis and mitochondrial stress induced by bleomycin, 64 mice were randomly divided into four groups. Control (Ctrl) group, GFW group, BLM group, GFW pretreatment and BLM exposure (B + G) group. In Ctrl group, mice were given the same dose of normal saline. In GFW group, mice were administered GFW (1 g/kg/day) *via* gavage before BLM exposure. The dose of GFW came from the existing literature (Ma et al., 2024). In BLM group, mice received a single intratracheal injection of BLM (5.0 mg/kg). The dose of BLM referred to our former investigation (Tong et al., 2021). In B + G group, mice were administered GFW (1 g/kg/day) *via* gavage starting 1 week before BLM injection and continued throughout the experiment period until tissue collection. ALL mice were euthanized at 7 days after BLM treatment.
(2) In Experiment two, to investigate the effects of GFW on BLM-induced IPF and EMT, 64 mice were divided into four groups as in Experiment one. In BLM and B + G groups, all mice were given a single intratracheal injection of BLM (5.0 mg/kg). In GFW and B + G groups, the mice were supplemented with GFW (1 g/kg/day) *via* gavage at 7 days before BLM injection and continued until the end of experiment (total 28 days). At 21 days after BLM exposure, all mice were euthanized and lung tissues were collected.

### 2.2 Cell culture and treatment

Human bronchial epithelial cells (BEAS-2B) were cultured in DMEM medium (Hyclone). This study encompassed two independent experiments. The first experiment aimed to investigate the impact of GFW on BLM-induced EMT in BEAS-2B cells. Cells were pretreated with GFW (100 μg/mL) for 24 h, followed by coculture with BLM (10 μg/mL) for an additional 48 h. The second experiment sought to examine the effect of GFW on BLM-induced ferroptosis and mitochondrial stress. Cells were pretreated with GFW (100 μg/mL) for 24 h, and then exposed to BLM (10 μg/mL). Lastly, cells were collected and relative markers were detected in 24 h after BLM exposure.

### 2.3 Dose selection

The animal dosing regimen was established based on the human clinical dose of Jiuzhitang GFW (2.7 g/day for adults, equivalent to 0.045 g/kg/day for a 60 kg individual). Following the body surface area (BSA) conversion method (Wu and Hu, 2008), the mouse equivalent dose was calculated. No adverse reactions were observed at this dose, and the administration volume was strictly controlled within 10 mL/kg to comply with animal welfare guidelines.

### 2.4 Western blotting

The total protein was extracted from BEAS-2B cells and mouse lungs by lysing buffer. The proteins underwent separation by SDS-PAGE gel electrophoresis and were subsequently transferred to the PVDF membrane. After blocking with Quick Blocking Solution, the membranes were incubated with primary antibodies and secondary antibodies for different times. The proteins expressions were detected using a chemiluminescent solution and analyzed with ImageJ software.

### 2.5 Histopathology and immunohistochemistry

Lung tissues were collected and fixed in 4% paraformaldehyde. After paraffin embedding, mouse lungs were cut into 4 µm sections and stained with hematoxylin and eosin (H&E). Pathological images were observed and captured using a microscope. Pathological analyses were conducted and the count of inflammatory cells were calculated. Collagen deposition was assessed using Masson’s trichrome staining and Sirius red staining (Li et al., 2024a). Moreover, the severity of fibrosis was assessed by Ashcroft score (Matute-Bello et al., 2011). For immunohistochemistry (IHC), paraffin sections were dewaxed, hydrated, and subjected to antigen repair in the boiling sodium citrate buffer. After blocking with goat serum for 2 h at 37°C, the sections were incubated with the relevant primary antibodies and secondary antibodies at room temperature. Then, a color development reaction was performed using DAB (3,3′-diaminobenzidine). Hematoxylin was used for nuclear staining. Finally, the target protein was examined and imaged using a light microscope for further analysis.

### 2.6 Enzyme-linked immunosorbent assays (ELISA)

Blood samples were drawing from eyeball in mice and separated at 3,500 rpm for 10 min. Then, serum samples were collected and then stored in an ultra-low temperature refrigerator for backup. The levels of SOD (MU30590, Bio-swamp), CAT (E-EL-0041, Elabscience), TGF-β1 (E-EL-0162 Elabscience), eight-epi-PGF2α (CSB-E10527m, cusabio Wuhan) and 8-OHdG (CSB-E14190m, cusabio Wuhan) were measured in serum samples using ELISA kits in accordance with our previous investigation (Wang et al., 2024).

### 2.7 Determination of MDA and GSH

Cells were harvested using RIPA lysis buffer, with 100 µL of lysis buffer added per one million cells. After spinning at 12,000 g for 10 min, we collected the supernatant. The MDA assay kit was obtained from Biyuntian Biotechnology Co., glutathione (GSH) kit was acquired from Nanjing Jianjian Biological Research Institute. The reagents were mixed and added. The absorbance was then measured after heating. An analogous procedure was adopted for the analysis in mouse serum. Lastly, the values of MDA, total glutathione (T-GSH) and glutathione disulfide (GSSG) were calculated.

### 2.8 Perl’s staining and iron content detection

Iron deposition was assessed in lung tissues using Perl’s staining (Li et al., 2022). Paraffin-embedded lung sections were deparaffinized and hydrated following standard protocols. The staining solution was added to the sections. After completing the reaction, the lung sections were sealed and examined with a microscope. To measure the concentration of Fe^2+^ in BEAS-2B cells, the Ferrorange assay kit (DOJINDO, Japan) was utilized. The cells were incubated with the working solution for 30 min. Subsequent analysis was performed using a fluorescence microscope.

### 2.9 Detection of hydroxyproline

BEAS-2B cells were inoculated in 100 mm Petri dishes and treated with BLM. Following the instructions of hydroxyproline kit (Nanjing Jiancheng Biological Research Institute, China), the tissue was ground and digested for 3 h at 37°C. The detection solution was added sequentially. An appropriate amount of the supernatant was collected to measure the absorbance at 550 nm.

### 2.10 Measurement of mitochondrial membrane potential (MMP) and ATP

To assess the MMP in BEAS-2B cells, JC-1 staining was utilized. After exposure to BLM, JC-1 staining solution was added and incubated for 20 min. Then, the supernatant was discarded. The stained cells were examined with fluorescence microscopy. ATP, a critical energy molecule, is served as an indicator of mitochondrial function (Li et al., 2022). After BLM incubation, BEAS-2B cells were lysed and the supernatant was collected. The level of ATP was detected using ATP kit (Beyotime Biotechnology, China). Then, the assay working solution was added and the ATP levels were quantified using a luminometer.

### 2.11 Statistical analysis

Normal-distributed data are expressed as mean ± standard error of the mean (SEM). Differences among four groups were analyzed by one-way ANOVA with Student–Newman–Keuls post hoc test. *P values* were adjusted via false discovery rate (FDR) using Benjamini–Hochberg correction for multiple comparisons. *P* < 0.05 was considered to represent statistical significance.

## 3 Results

### 3.1 Supplementation with GFW mitigated BLM-induced lung injury

**FIGURE 1:**
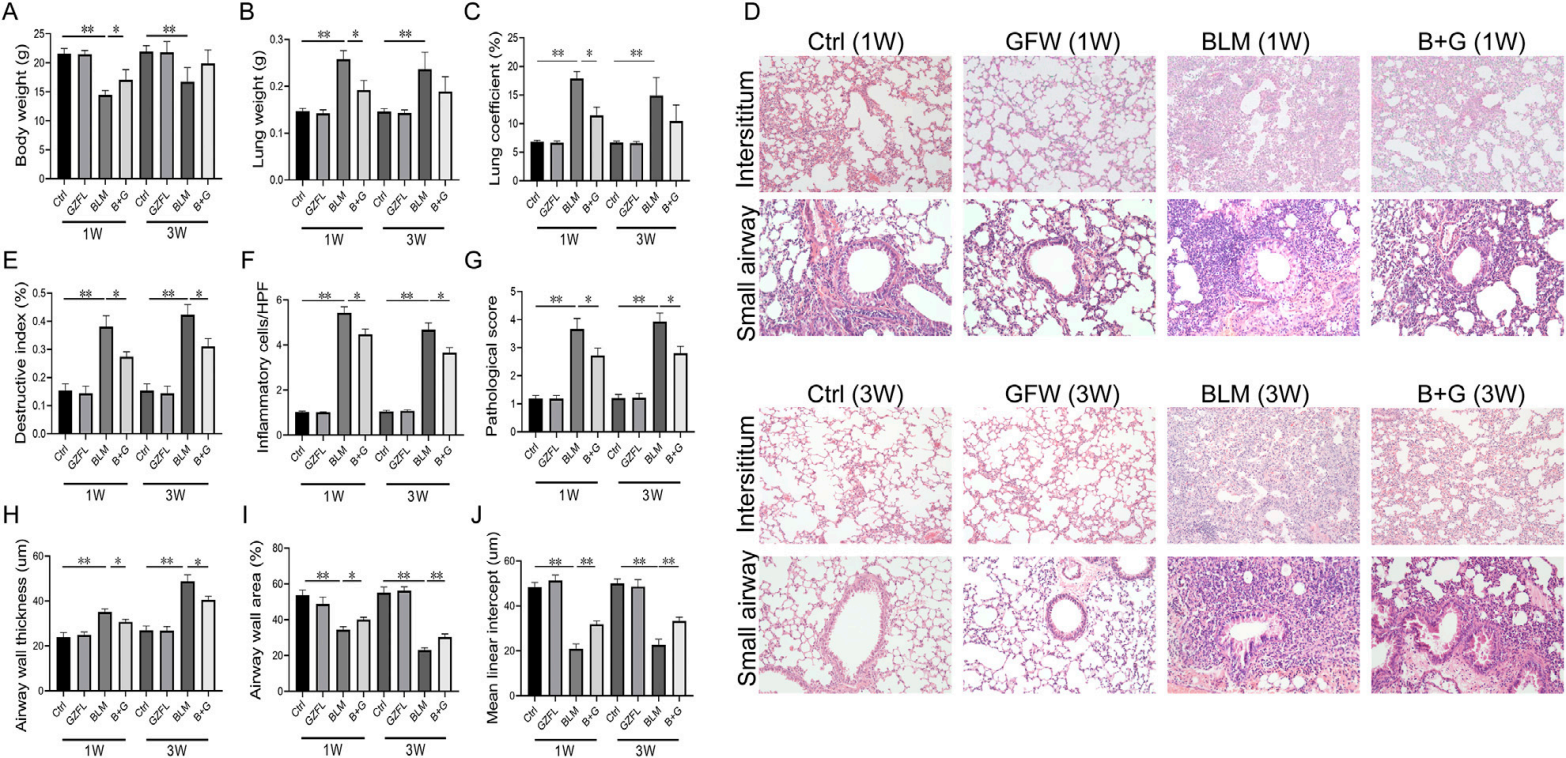
GFW supplementation attenuated BLM-induced lung injury in mice. Balb/c mice were intratracheally instilled with BLM (5 mg/kg) with or without GFW (1 g/kg) pretreatment. Lung specimens were collected in 7 and 21 days after BLM exposure. The effect of GFW supplementation on BLM-induced lung injury was assessed in mice lungs **(A)** Body weight **(B)** Lung weight **(C)** Lung coefficient **(D)** Lung interstitium and airway histopathological damage were evaluated by HE staining. Scale bar: 100 µm. Original magnification: ×100 **(E)** Destructive index **(F)** The count of inflammatory cells per high-power field (HPF) **(G)** Pathological score **(H)** Airway wall thickness **(I)** Airway wall area **(J)** Mean linear intercept. All data were expressed as means ± S.E.M. (N = 15). *P < 0.05, **P < 0.01.

The effect of GFW supplementation on BLM-induced lung injury was evaluated in mice. As shown Figures 1A–C, body weight was increased, but lung weight and lung coefficient were decreased in 1 week and 3 weeks after BLM injection. Although GFW supplementation had no effect of BLM-induced the changes of body weight, lung weight, and lung coefficient in 3 weeks. The alternations of body weight, lung weight, and lung coefficient were obviously restored by GFW supplementation in 1 week after BLM injection. In addition, BLM exposure evoked alveolar structure destruction, inflammatory cell infiltration, interstitial damage, and airway constriction in mouse lungs (Figure 1D). Amazingly, GFW pretreatment significantly alleviated BLM-induced lung injury and lung fibrosis (Figure 1D). Additionally, destructive index, inflammatory cells, airway wall thickness, and pathological score were upregulated in BLM-injected mice. On the contrary, BLM exposure inhibited airway wall area and mean linear intercept in mouse lungs (Figures 1E–J). As expected, supplementation with GFW significantly attenuated BLM-incurred the damage of alveoli and interstitium in mouse lungs (Figures 1E–J).

### 3.2 Supplementation with GFW alleviated BLM-evoked fibrosis in mouse lungs

**FIGURE 2:**
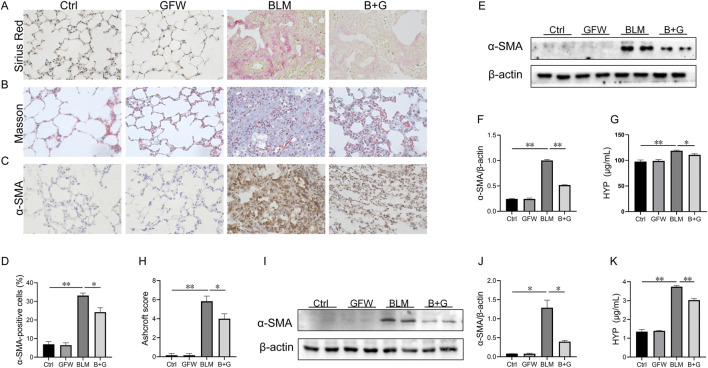
GFW supplementation alleviated BLM-evoked pulmonary fibrosis in mouse lungs. Balb/c mice were intratracheally instilled with BLM (5 mg/kg) with or without GFW (1 g/kg) pretreatment. Lung specimens were collected in 7 and 21 days after BLM exposure. The effect of GFW supplementation on BLM-induced pulmonary fibrosis was estimated in mice **(A)** Collagen deposition was analyzed by Sirius Red staining **(B)** Collagen deposition was estimated by Masson’s trichrome staining **(C)** α-SMA-positive cells were evaluated by IHC **(D)** The number of α-SMA positive cells was calculated **(E)** The protein expression of α-SMA was detected in lung tissues by Western blotting **(F)** Quantification analysis of α-SMA was conducted **(G)** Hydroxyproline content was detected in lung tissues **(H)** The severity degree of fibrosis was estimated by Ashcroft score **(I)** The protein expression of α-SMA was detected in BEAS-2B cells by Western blotting **(J)** Quantification analysis of α-SMA expression was conducted **(K)** Hydroxyproline content was detected in BEAS-2B cells. All data were expressed as means ± S.E.M. (N = 6). *P < 0.05, **P < 0.01.

To study the impact of GFW on BLM-induced collagen deposition, Masson staining and Sirius red staining were performed in 3 weeks after BLM treatment. A significant amount of collagen was deposited in the lung interstitium after BLM exposure (Figures 2A, B). As expected, pretreatment with GFW obviously reduced BLM-triggered collagen deposition in lung tissues of mice (Figures 2A, B). Moreover, the positive cells and protein expression of alpha smooth muscle actin (α-SMA), another marker of fibrosis, were elevated in lung tissues after BLM exposure (Figures 2C–F). GFW supplementation downregulated BLM-caused elevation of α-SMA in mouse lungs (Figures 2C–F). Besides, the content of hydroxyproline (HYP) was measured in lung tissues. The results indicated that pulmonary hydroxyproline was increased in BLM-injected mice. Meanwhile, supplementation with GFW significantly repressed BLM-evoked upregulation of pulmonary hydroxyproline (Figure 2G). As shown in Figure 2H, GFW supplementation downregulated BLM-mediated upregulation of Aschroft score in lung tissues. As shown in Figures 2I,J, GFW incubation repressed BLM-incurred α-SMA increase in BEAS-2B cells. Lastly, GFW pretreatment significantly inhibited BLM-induced elevation of HYP in BEAS-2B cells (Figure 2K).

### 3.3 Supplementation with GFW attenuated BLM-induced EMT in mouse lungs and BEAS-2B cells

**FIGURE 3:**
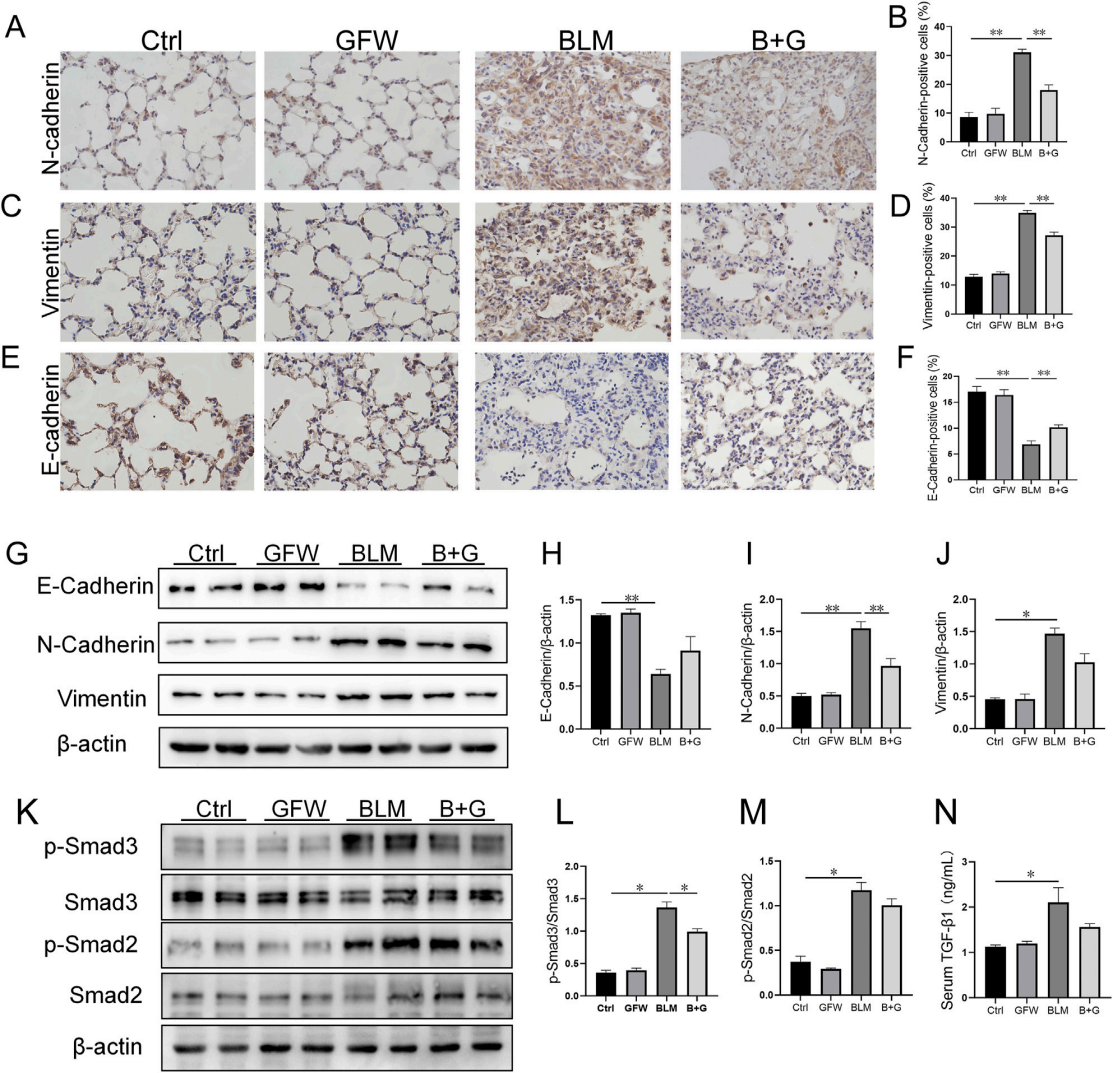
GFW supplementation relived BLM-excited EMT in mouse lungs. Balb/c mice were intratracheally instilled with BLM (5 mg/kg) with or without GFW (1 g/kg) pretreatment. Lung specimens were collected in 7 and 21 days after BLM exposure **(A–J)** The effect of GFW supplementation on BLM-induced EMT was estimated in mice lungs **(A)** N-cadherin-positive cells was detected by IHC **(B)** The number of N-cadherin-positive cells was calculated **(C)** Vimentin-positive cells were detected by IHC **(D)** The number of Vimentin-positive cells was calculated **(E)** E-cadherin-positive cells was detected by IHC **(F)** The number of E-cadherin-positive cells was calculated **(G)** The markers of EMT were measured by Western blotting **(H–J)** Quantification analyses of proteins expression were conducted **(H)** E-cadherin **(I)** N-cadherin **(J)** Vimentin **(K–N)** The influence of GFW supplementation on BLM-activated TGF-β/Smad signaling was detected in mouse lungs **(K)** The expressions of Smads and phosphorylated Smads were measured using Western blotting **(L)** p-Smad3/Smad3 **(M)** p-Smad2/Smad2 **(N)** The level of serum TGF-β1 was detected using ELISA. All data were expressed as means ± S.E.M. (N = 6). *P < 0.05, **P < 0.01.

The influence of GFW supplementation on BLM-induced pulmonary EMT was explored in 3 weeks after BLM injection. As shown in Figures 3A–D, the number of N-cadherin- and Vimentin-positive cells, two markers of mesenchyme, were increased in BLM-injected mouse lungs. By contrast, the count of E-cadherin-positive cells, a hallmark of epithelium, was decreased in mouse lungs after BLM exposure. Amusingly, GFW supplementation dominantly inhibited BLM-caused decline of E-cadherin in lung tissues (Figures 3E, F). In addition, the impact of GFW incubation on BLM-triggered EMT was analyzed in BEAS-2B cells. As shown in Figures 3G–J, GFW incubation prominently attenuated BLM-induced decline of E-cadherin, and elevation of N-cadherin and Vimentin in BEAS-2B cells. In addition, TGF-β1/Smad signaling was analyzed. The results demonstrated that GFW supplementation obviously alleviated the phosphorylation level of Smad 3 (Figure 3K–M). Besides, the concentration of serum TGF-β1 was upregulated in mice after BLM exposure. There was no obvious effect of GFW supplementation on BLM-upregulated serum TGF-β1 (Figure 3N).

### 3.4 Supplementation with GFW ameliorated BLM-triggered ferroptosis in mouse lungs

**FIGURE 4:**
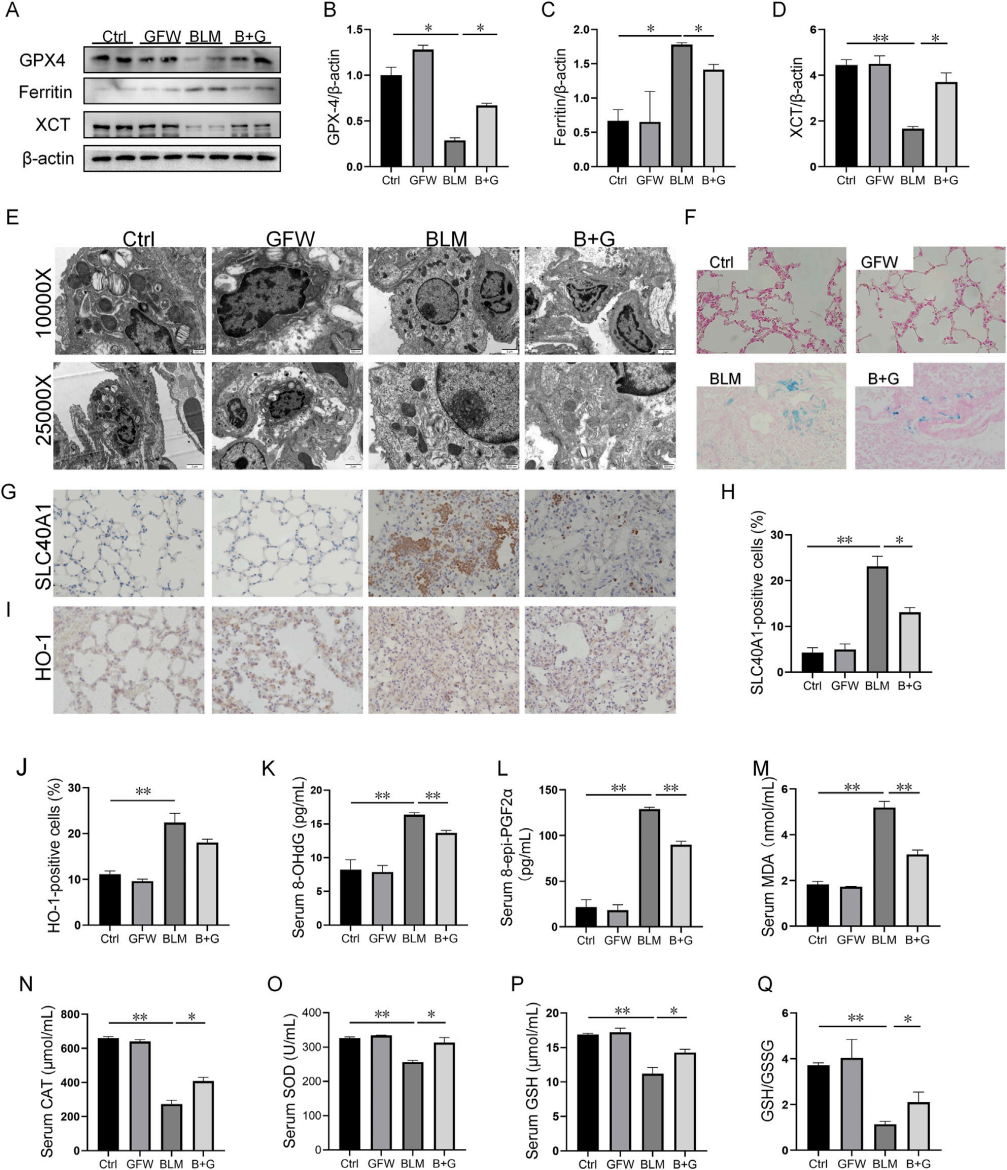
GFW supplementation mitigated BLM-provoked ferroptosis in mice lungs. Balb/c mice were intratracheally instilled with BLM (5 mg/kg) with or without GFW (1 g/kg) pretreatment. Lung specimens were collected in 7 and 21 days after BLM exposure. The effect of GFW supplementation on BLM-induced ferroptosis was estimated in mice lungs **(A)** The markers of ferroptosis were determined via Western blotting **(B–D)** Quantitative analyses of proteins expression were assessed **(B)** GPX4 **(C)** Ferritin **(D)** XCT **(E)** Mitochondrial morphology was observed using transmission electron microscopy **(F)** Iron deposition was evaluated using Perl’s staining **(G, H)** SLC40A1-positive cells were measured using IHC **(I, J)** HO-1-positive cells were measured using IHC **(K–N)** The levels of lipid peroxidation parameters were measured in serum using ELISA **(K)** 8-OHdG **(L)** eight-epi-PGF2α **(M)** MDA **(N)** CAT **(O–Q)** The contents of oxidative stress indices were measured in serum by biochemical methods **(O)** SOD **(P)** GSH **(Q)** The ratio of GSH/GSSG. All data were expressed as means ± S.E.M. (N = 6). *P < 0.05, **P < 0.01.

The effect of GFW supplementation on BLM-incurred ferroptosis was explored in mouse lungs. The results indicated that the proteins expressions of GPX4 and XCT were reduced, and ferritin expression was elevated in 1 week after BLM exposure (Figures 4A–D). However, GFW supplementation inhibited BLM-evoked alternations of GPX4, XCT, and ferritin in mouse lungs (Figures 4A–D). Additionally, the mitochondrial morphology was observed in lung tissues by transmission electron microscopy. After BLM treatment, mitochondrial membranes were fractured, densities were increased, and mitochondrial cristae was decreased and disappeared in pulmonary epithelial cells of lung tissues (Figure 4E). However, GFW supplementation ameliorated BLM-mediated mitochondrial damage and ultrastructural disruption in pulmonary epithelial cells of lung tissues. In addition, the deposition of Fe^2+^ was detected in lung sections through Prussian blue staining. The results suggested that GFW supplementation attenuated BLM-incurred iron deposition in mouse lungs (Figure 4F). Meanwhile, GFW supplementation repressed BLM-upregulated SLC40A1 in mouse lungs (Figures 4G, H). Then, the influence of GFW supplementation on BLM-induced lipid peroxidation was estimated in mice lungs. In BLM group, the number of HO-1-positive cells was increased (Figures 4I, J), the levels of 8-OHdG, eight-iso-PGF2α, and MDA were elevated in mice lungs. In addition, the contents of SOD, CAT, GSH, the ratio of GSH to GSSG, were all declined in mouse lungs (Figure 4H–Q). As expected, GFW supplementation obviously alleviated BLM-evoked lipid peroxidation in mouse lungs (Figure 4I–Q).

### 3.5 Pretreatment with GFW relieved BLM-excited ferroptosis in BEAS-2B cells

**FIGURE 5:**
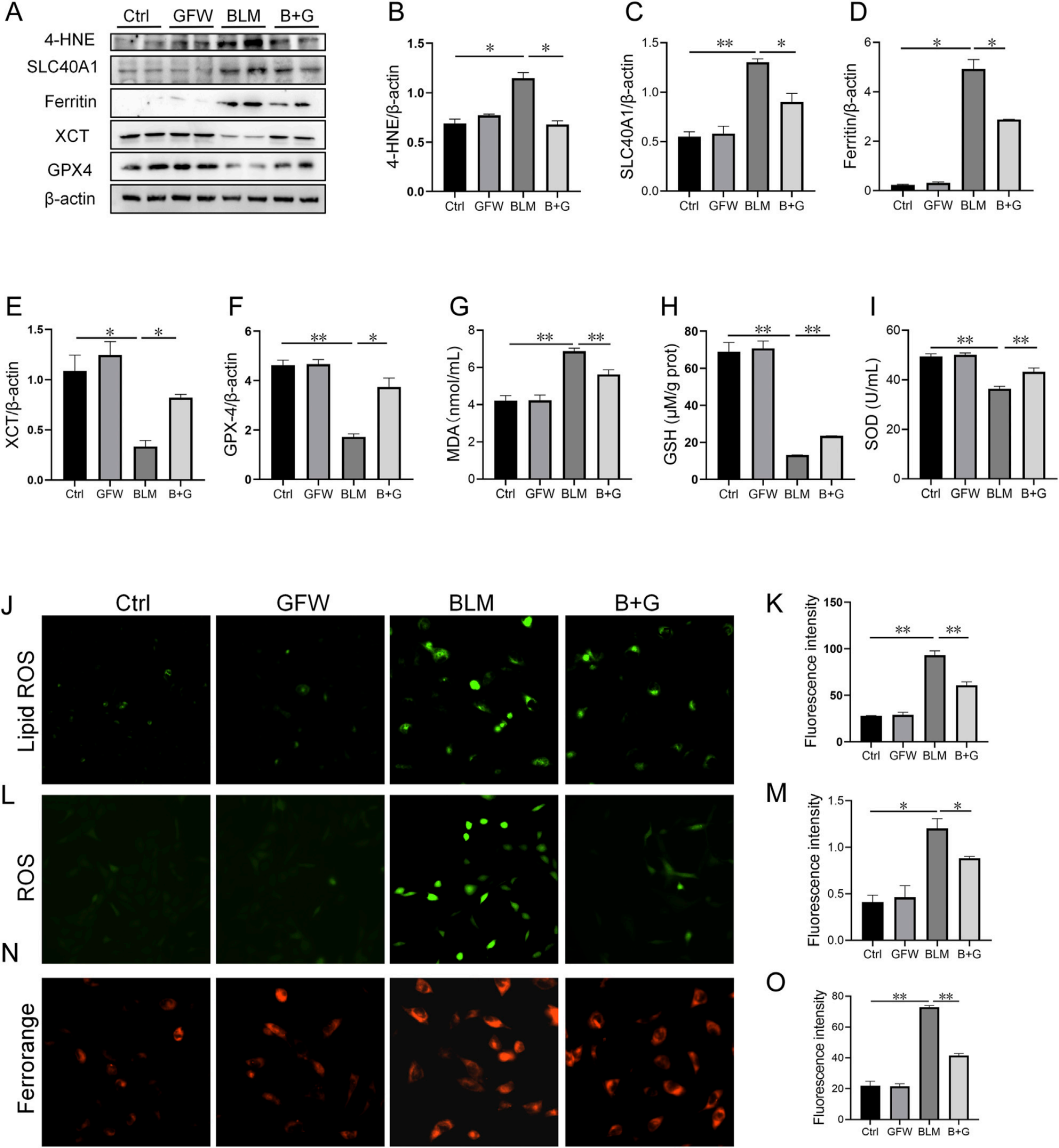
GFW pretreatment abated BLM-caused ferroptosis in BEAS-2B cells. BEAS-2B cells were exposed to BLM (10 μg/mL) for 24 h with or without GFW (100 μg/mL) coculture. The effect of GFW coculture on BLM-caused ferroptosis was analyzed in BEAS-2B cells **(A)** The markers of ferroptosis were detected *via* Western blotting **(B–E)** Quantitative analyses of proteins expression were assessed **(B)** 4-HNE **(C)** SLC40A1 **(D)** Ferritin **(E)** XCT **(F)** GPX4 **(G–I)** The levels of lipid peroxidation were determined in BEAS-2B cells **(G)** MDA **(H)** GSH **(I)** SOD **(J)** Lipid ROS was determined in BEAS-2B cells **(K)** Fluorescence intensity of lipid ROS was analyzed **(L)** ROS was evaluated in BEAS-2B cells **(M)** Fluorescence intensity of ROS was analyzed **(N)** Ferrorange stain was conducted in BEAS-2B cells **(O)** Quantitative analysis of Ferrorange was performed. All data were expressed as means ± S.E.M. (N = 6). *P < 0.05, **P < 0.01.

The effect of GFW on BLM-induced ferroptosis was explored in BEAS-2B cells. The results suggested that the proteins expressions of 4-HNE, SLC40A1, and ferritin were elevated, XCT and GPX4 protein expression were declined in BLM-treated BEAS-2B cells (Figures 5A–F). As expected, pretreatment with GFW obviously restored these changes of proteins expressions (Figures 5A–F). In addition, pretreatment with GFW evidently alleviated BLM-induced upregulation of MDA in BEAS-2B cells (Figure 5G G). On the contrary, GFW coculture effectively repressed BLM-incurred downregulation of GSH and SOD in BEAS-2B cells (Figures 5H, I). After BLM exposure, lipid ROS and ROS were elevated, while GFW pretreatment reduced the production of lipid ROS and ROS induced by BLM in BEAS-2B cells (Figure 5J-M). Moreover, Ferrorange probe indicated that GFW pretreatment significantly inhibited BLM-mediated the increase of intracellular Fe^2+^ level in BEAS-2B cells (Figures 5N, O).

### 3.6 Pretreatment with GFW repressed BLM-provoked mitochondrial stress in mouse lungs and BEAS-2B cells

**FIGURE 6:**
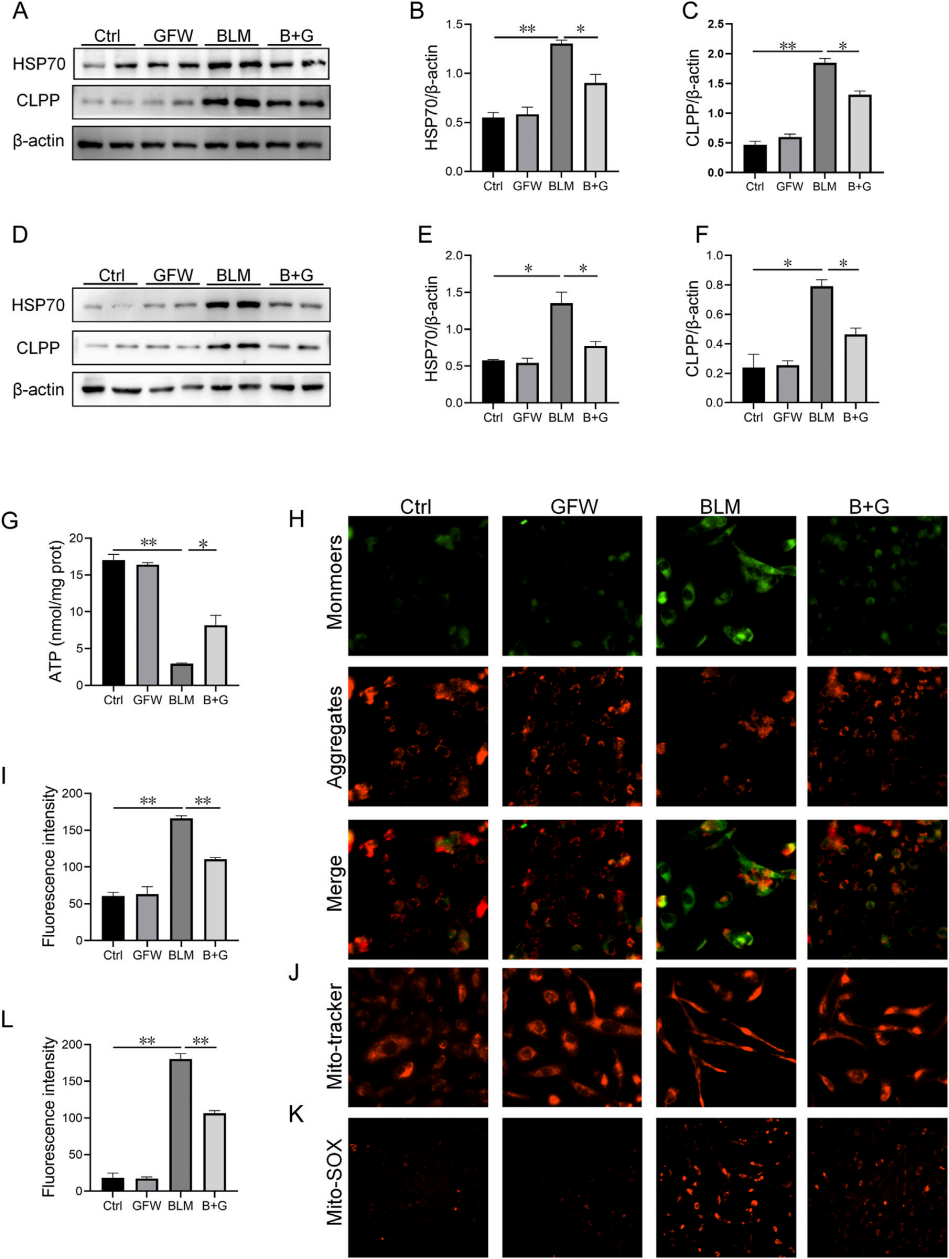
GFW supplementation abolished BLM-caused mitochondrial stress in mouse lungs and BEAS-2B cells. Balb/c mice were intratracheally instilled with BLM (5 mg/kg) with or without GFW (1 g/kg) pretreatment. Lung specimens were collected in 7 and 21 days after BLM exposure. The effect of GFW supplementation on BLM-induced mitochondrial stress was assessed in mice lungs **(A)** The proteins expressions of HSP70 and CLPP were detected using Western blotting **(B, C)** Quantitative analyses were conducted **(B)** HSP70 **(C)** CLPP **(D–K)** BEAS-2B cells were exposed to BLM (10 μg/mL) for 24 h with or without GFW (100 μg/mL) pretreatment **(D)** The markers of mitochondrial stress were measured using Western blotting **(E, F)** Quantitative analyses were performed **(E)** CLPP **(F)** HSP70 **(G)**The content of ATP was measured **(H)** MMP was detected through JC-1 staining **(I)** Quantitative analysis of MMP was conducted **(J)** MMP was detected by MitoTracker Red CMXRos **(K)** MtROS were measured by MitoSOX™ Red staining **(L)** Quantitative analysis of mtROS was performed. All data were expressed as means ± S.E.M. (N = 6). *P < 0.05, **P < 0.01.

The influences of GFW supplementation on BLM-excited mitochondrial stress was explored *in vivo* and *in vitro* experiments. As shown in Figures 6A–C, BLM treatment evoked the increase of HSP70 and CLPP in mice lungs. Interestingly, supplementation with GFW inhibited elevation of HSP70 and CLPP induced by BLM (Figures 6A–C). Similarly, GFW coculture repressed BLM-induced upregulation of HSP70 and CLPP in BEAS-2B cells (Figures 6D–F). Additionally, the amount of ATP, which represents the functional of cellular mitochondria, was significantly decreased in BEAS-2B cells after BLM exposure. GFW pretreatment partially restored BLM-induced decline of ATP content (Figure 6G). Moreover, the MMP was evaluated via MitoTracker Red CMXRos and JC-1 staining. GFW pretreatment attenuated BLM-induced downregulation of MMP in BEAS-2B cells (Figures 6H–J). Additionally, mitochondrial reactive oxygen species (mtROS) was detected using Mito-SOX probe. As shown in Figures 6K, L, the production of mtROS was obviously elevated in BLM-exposed BEAS-2B cells. As expected, GFW pretreatment repressed BLM-incurred elevation of mtROS in BEAS-2B cells (Figure 6K, L).

## 4 Discussion

In this study, the impact of GFW supplementation on pulmonary fibrosis induced by BLM was explored in mouse lungs and the potential mechanisms were analyzed in pulmonary epithelial cells. Our results indicated that: (1) GFW supplementation alleviated BLM-induced lung injury and pulmonary fibrosis; (2) GFW supplementation mitigated BLM-provoked EMT in mice lungs; (3) GFW supplementation attenuated BLM-excited ferroptosis in mouse lungs and pulmonary epithelial cells; (4) GFW supplementation relieved mitochondrial stress in mouse lungs and pulmonary epithelial cells. These results provided experimental evidence that pretreatment with GFW can effectively inhibit BLM-evoked pulmonary fibrosis.

In Traditional Chinese Medicine (TCM) theory, IPF is associated with qi stagnation and blood stasis, and its treatment requires regulating qi and blood. (Suxian et al., 2018). GFW has the functions of activating blood circulation to remove blood stasis and gradually reducing hard lumps. In the formula, Cinnamon Twig (Guizhi) serves as the principal herb to warm and unblock the blood vessels and eliminate stasis. In addition, Peach Seed (Taoren) acts as the secondary herb to activate blood and remove stasis. Tree Peony Bark (Mudanpi) and Peony Root (Shaoyao) also help activate blood and resolve stasis, while Poria (Fuling) strengthens the spleen and boosts energy. Therefore, GFW may be suitable for the treatment of IPF. The previous clues have suggested that GFW may alleviate the progress of liver fibrosis. Therefore, we explored the protective effect of GFW supplementation on BLM-incurred pulmonary fibrosis in mice lungs. These results found that supplementation with GFW effectively repressed BLM-induced lung injury and inflammatory cell infiltration in mice lungs. In addition, BLM-evoked collagen deposition was obviously repressed in mouse lungs by GFW supplementation. Therefore, these results revealed that supplementation with GFW obviously alleviated BLM-induced lung injury and pulmonary fibrosis.

The process of epithelial to mesenchymal cell differentiation is known as EMT (Guo et al., 2015). EMT has a vital function in multiple biological processes, such as embryonic development, tumor migration, and fibrosis in different organs (Lee and Massagué, 2022). The previous investigation has demonstrated that EMT has been shown to be involved in the development of IPF (Chapman, 2011). In addition, BLM exposure results in pulmonary fibrosis through inducing EMT in alveolar epithelial cells (Tanjore et al., 2009). The differentiation of epithelial cells into myofibroblasts, can acquire migratory and invasive properties, and secrete excessive amounts of extracellular matrix in myofibroblasts (Yan et al., 2024). Inhibition of EMT ameliorates the progression of pulmonary fibrosis (Liu W. et al., 2022). So, the effect of GFW supplementation on BLM-evoked EMT was evaluated in mice. Consistent with previous results, E-cadherin, the marker of epithelium, was reduced in BLM-exposed mice lungs. Besides, the expressions of N-cadherin and Vimentin, two hallmarks of mesenchyme, were elevated in lung mice after BLM exposure. Surprisingly, GFW supplementation obviously ameliorated BLM-provoked EMT in mice lungs. The previous studies have TGF-β/Smad signaling activation can regulate EMT (Fei et al., 2019; Fu et al., 2021). Previous research has demonstrated that BLM activates TGF-β1, a key regulator of ECM accumulation and EMT in IPF (Zhang et al., 2023). Smad2 and Smad3 are the primary downstream mediators of TGF-β. Upon activation of TGF-β signaling, cytoplasmic Smads undergo phosphorylation, and the phosphorylated Smad2/Smad3 complex translocate to the nucleus to regulate transcription of target genes and regulate EMT pathway (Nakamura et al., 2023). As expected, GFW supplementation markedly repressed the phosphorylation of Smad3 in mouse lungs. However, there was no dramatic effect of GFW supplementation on BLM-induced increase of serum TGF-β. Thus, we speculated that GFW supplementation inhibites EMT in mouse lungs via not TGF-β/Smad signaling. Maybe there are other regulatory mechanisms mediating EMT. Therefore, GFW supplementation alleviates BLM-excited pulmonary fibrosis partially through inhibiting EMT in mouse lungs.

Ferroptosis, a novel type of cellular death, is identified in recent years. Ferroptosis is characterized by the disruption of cell membranes and cell death driven by the iron-dependent accumulation of lipid peroxides (Stockwell, 2022). The morphological features of ferroptosis indicate the loss of mitochondrial cristae, the rupture of the outer membrane, and a decrease in mitochondrial volume (Kazan and Kalaipandian, 2019). Additionally, it is accompanied by a reduction of glutathione and an accumulation of oxidized glutathione (glutathione disulfide), and iron overload in the process of ferroptosis (Liu Y. et al., 2022). More and more studies have suggested that pulmonary fibrosis is strongly associated with iron deposition and lipid peroxidation (He et al., 2022; Wang et al., 2023). An earlier study confirmed that BLM exposure induces ferroptosis in mouse lungs and BEAS-2B cells (Zhan et al., 2022). Interestingly, the application of ferroptosis inhibitor sensibly relieves BLM-mediated pulmonary fibrosis in mice model (Pei et al., 2022). These data strongly highlighted the possibility of mitigating ferroptosis as a treatment approach of pulmonary fibrosis. In our study, we found that the increase of ferritin and the decrease of GPX4 induced by BLM in mouse lungs and BEAS-2B cells were prominently inhibited by GFW pretreatment. In addition, BLM-provoked iron overload and lipid peroxidation in lungs were significantly alleviated in GFW-supplemented mice and BEAS-2B cells. So, GFW supplementation attenuates BLM-evoked pulmonary fibrosis partially through repressing ferroptosis in pulmonary epithelial cells.

Mitochondria serve as a crucial function in regulating cell death and is a major source of ROS (Orrenius et al., 2007). Furthermore, mitochondrial stress is implicated in the development of pulmonary fibrosis (Lee et al., 2024). BLM exposure has been found to induce a significant increase in the production of mtROS and mitochondrial DNA (mtDNA) (Cloonan, 2017). Excessive production of mtROS further disrupt the balance of the antioxidant system, consequently impacting ATP production and losing mitochondrial membrane potential. The impairment of mitochondrial function not only contributes to severe cellular damage but also triggers myofibroblasts activation, thereby promoting the progression of pulmonary fibrosis (Liu et al., 2023). The previous research suggested that BLM exposure incurs mitochondria stress in mouse lungs and pulmonary epithelial cells (Zhan et al., 2022). Sheat shock protein 70 (HSP70) and CLPP protease are crucial proteins involved in maintaining cellular and mitochondrial homeostasis (Ambrose and Chapman, 2021). According to our study findings, HSP70 and CLPP, two markers of mitochondrial stress, were increased in mouse lungs and BEAS-2B cells after BLM exposure. Interestingly, supplementation with GFW relieved BLM-induced upregulation of HSP70 and CLPP. Furthermore, pretreatment with GFW reduced BLM-evoked the production of mtROS, and partially restored the elevation of MMP and ATP in pulmonary epithelial cells. Although the role of mitochondria in ferroptosis remains controversial, it has been observed that the targeted mitochondrial antioxidants effectively inhibit ferroptosis in human pulmonary epithelial cells (Zhan et al., 2022). Additionally, excessive mtROS initiates ferroptosis via regulating the iron homeostasis (Wei et al., 2020). Therefore, this may be one of the ways in which GFW can mitigate BLM-evoked ferroptosis. Collectively, GFW supplementation relieves BLM-incurred pulmonary fibrosis partially through attenuating mtROS-initiated ferroptosis in pulmonary epithelial cells.

## 5 Conclusion

In summary, the current research mainly analyzed the effect of GFW supplementation on BLM-evoked pulmonary fibrosis and potential mechanisms. Our data indicates that GFW supplementation obviously alleviates BLM-induced lung injury and pulmonary fibrosis. Mechanistically, supplementation with GFW improves BLM-incurred lung injury and pulmonary fibrosis partially through repressing EMT and mtROS-initiated ferroptosis in pulmonary epithelial cells. Our research provided experimental evidences that GFW may be used as a potentially preventive and therapeutic drugs for BLM-incurred lung injury and pulmonary fibrosis.

## Data Availability

The raw data supporting the conclusions of this article will be made available by the authors, without undue reservation.
